# Supplementary material for: Hyperexcitable but seizure-free: hippocampal network dysfunction in a Wolfram syndrome model
Source: bioRxiv. 2026 Oct 2:2026.09.27.754840. Preprint. [Version 1] doi: 10.64898/2026.09.27.754840 (PMC13648544; doi:10.64898/2026.09.27.754840)
Supplement: Supplementary file 1 [file NIHPP2026.09.27.754840v1-supplement-1.pdf]

bioRxiv preprint doi: https://doi.org/10.64898/2026.09.27.754840; this version posted October 2, 2026. The copyright holder for this preprint (which was not certified by peer review) is the author/funder, who has granted bioRxiv a license to display the preprint in perpetuity. It is made available under a CC-BY 4.0 International license.

Supplementary Figures:

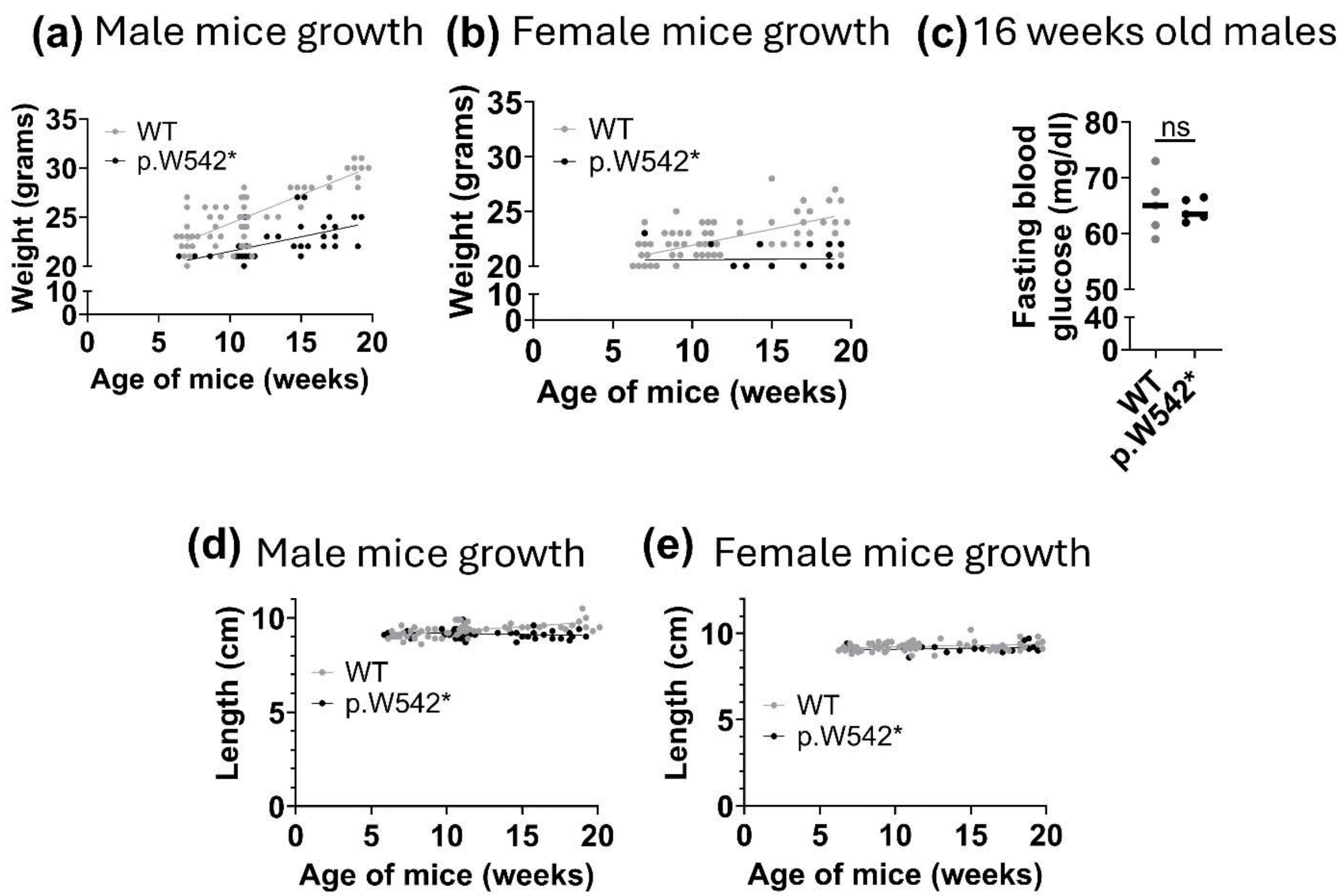

Supplementary figure 1: Reduced body weight gain in *Wfs1* p.W542 mice without fasting hyperglycemia. **(a, b)** Body weight from 6-20 weeks of age in male and female mice, with grey lines representing wild-type (WT) and black lines representing *Wfs1* p.W542* homozygous mice. Each data point represents a single animal. Males (WT n = 78, p.W542* = 38), females (WT n = 86, p.W542* = 29). Genotype effects were estimated by linear regression with age as a covariate and standard errors clustered on dam. Homozygous *Wfs1* p.W542* mice were lighter than WT in both sexes. (a) Males weight difference of −3.65 g (95% CI −4.91 to −2.39; p = 1.3 × 10□□) and (b) females, weight difference of −4.07 g (−5.15 to −2.98; p = 2.1 × 10□¹³). **(c)** Fasting blood glucose in 16-week-old males measured using a glucometer. Each data point is a different animal. Mann-Whitney test with $p > 0.99$. **(d-e)** Naso-anal length of mice against age (6-20 weeks), with each data point representing a different animal. Genotype effects were estimated by linear regression with age as a covariate and standard errors clustered on dam. Homozygous *Wfs1* p.W542* mice were shorter than WT in both sexes. (d) males length −0.38 cm (−0.53 to −0.23; p = 7.7 × 10□□) (e) females length −0.39 cm (−0.54 to −0.24; p = 3.1 × 10□□).

bioRxiv preprint doi: https://doi.org/10.64898/2026.09.27.754840; this version posted October 2, 2026. The copyright holder for this preprint (which was not certified by peer review) is the author/funder, who has granted bioRxiv a license to display the preprint in perpetuity. It is made available under a CC-BY 4.0 International license.

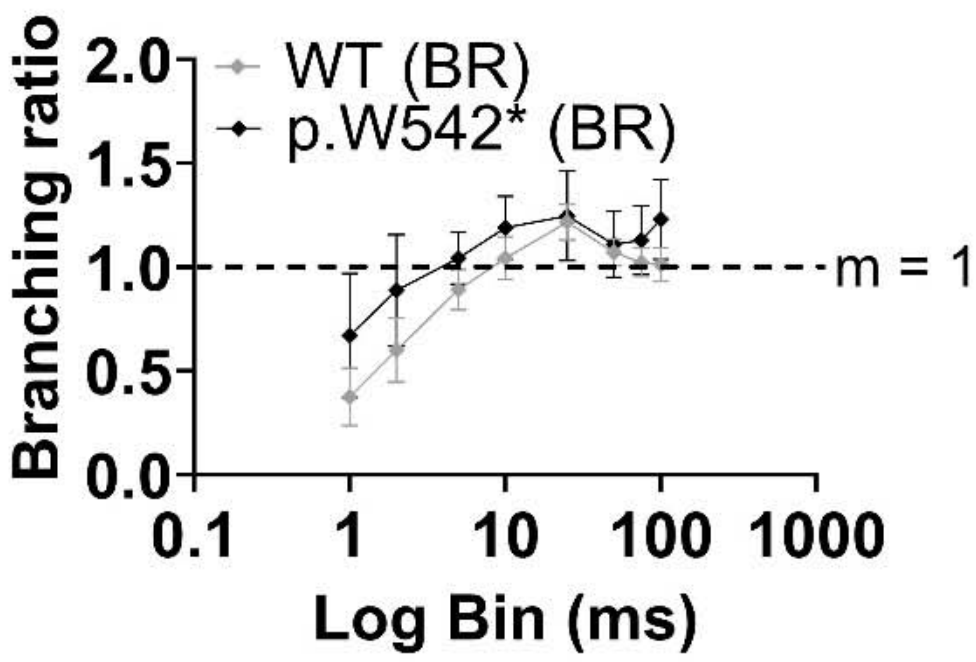

Supplementary figure 2: **The conventional single-step branching ratio estimator is bin-width dependent and does not resolve a genotype difference**. Branching ratio (BR) estimated by the conventional single-step method as a function of analysis bin width (1–100 ms, log scale) for wild-type (grey, n = 4) and Wfs1 p.W542* homozygous (black, n = 4) male hippocampal cultures at DIV14. Datapoints represent mean +/-SD. BR estimates rise monotonically with bin width in both genotypes and cross m = 1 at intermediate bins (20–50 ms), a known consequence of single step branching estimates[42], and spatial subsampling[43], rather than a change in the underlying network state.

bioRxiv preprint doi: https://doi.org/10.64898/2026.09.27.754840; this version posted October 2, 2026. The copyright holder for this preprint (which was not certified by peer review) is the author/funder, who has granted bioRxiv a license to display the preprint in perpetuity. It is made available under a CC-BY 4.0 International license.

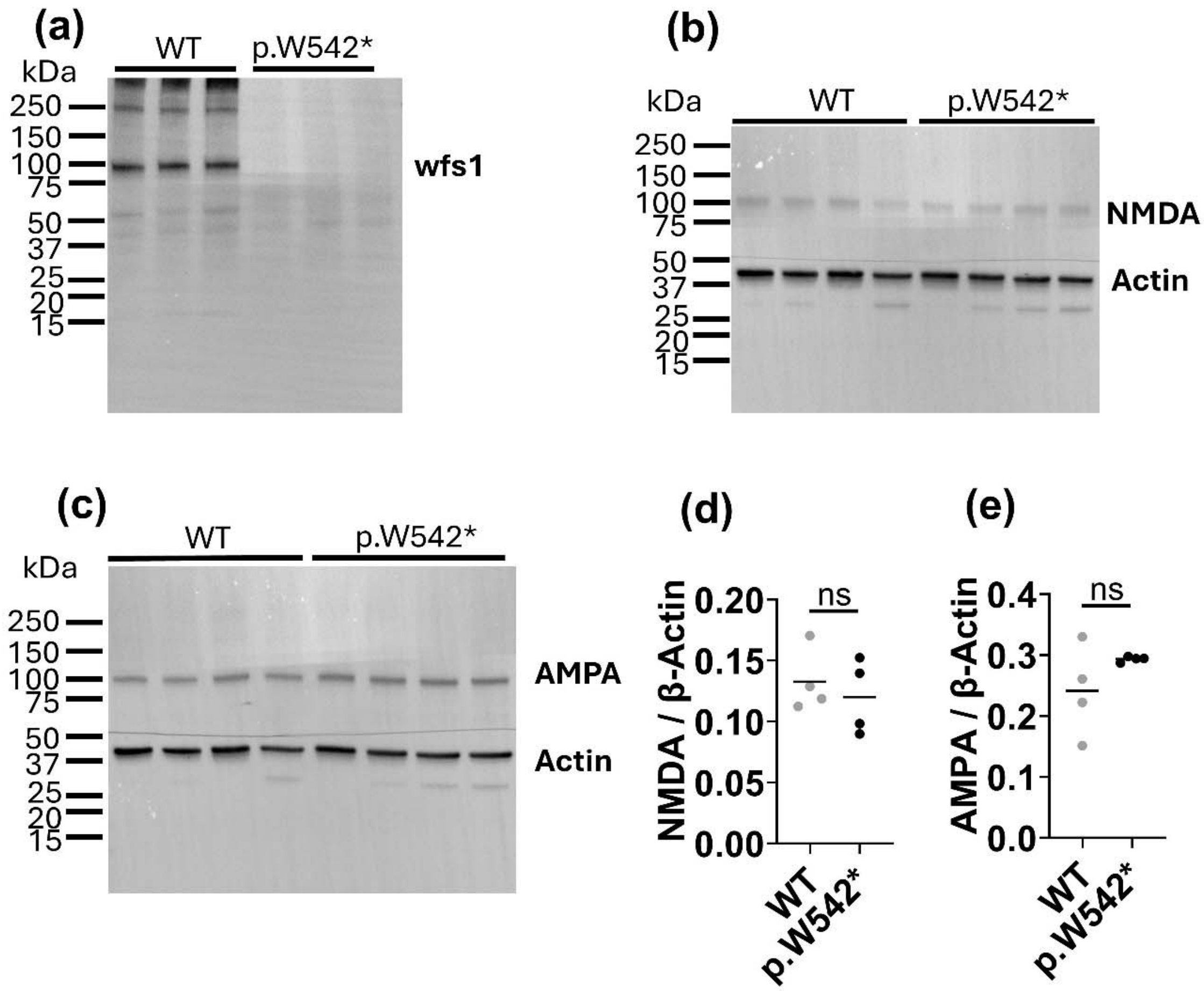

Supplementary Figure 3: Hippocampal protein expression of WFS1, NMDA and AMPA receptor expression in WT and *Wfs1* p.W542* homozygous male mice. **(a-c)** Representative full western blots showing (a) wfs1 (cropped image shown in figure 1) (b) NMDA receptor 1 (GluN1) (c) AMPA receptor 1 (GluA1). Each sample lane represents a separate male animal hippocampus. β-Actin was used as loading control. Molecular mass markers are indicated in kilodaltons (kDa). **(d, e)** Densitometric quantification of (d) NMDA receptor and (e) AMPA receptor band intensities, normalized to the corresponding β-actin signal. Gray symbols indicate WT samples, and black symbols indicate p.W542* homozygous male samples. Each point represents a separate male animal. Horizontal lines indicate mean. Statistical analyses was performed using Mann-Whitney test, and ns represents non-significant change with panel (d) $p = 0.68$, and panel (e) $p = 0.34$.

bioRxiv preprint doi: https://doi.org/10.64898/2026.09.27.754840; this version posted October 2, 2026. The copyright holder for this preprint (which was not certified by peer review) is the author/funder, who has granted bioRxiv a license to display the preprint in perpetuity. It is made available under a CC-BY 4.0 International license.

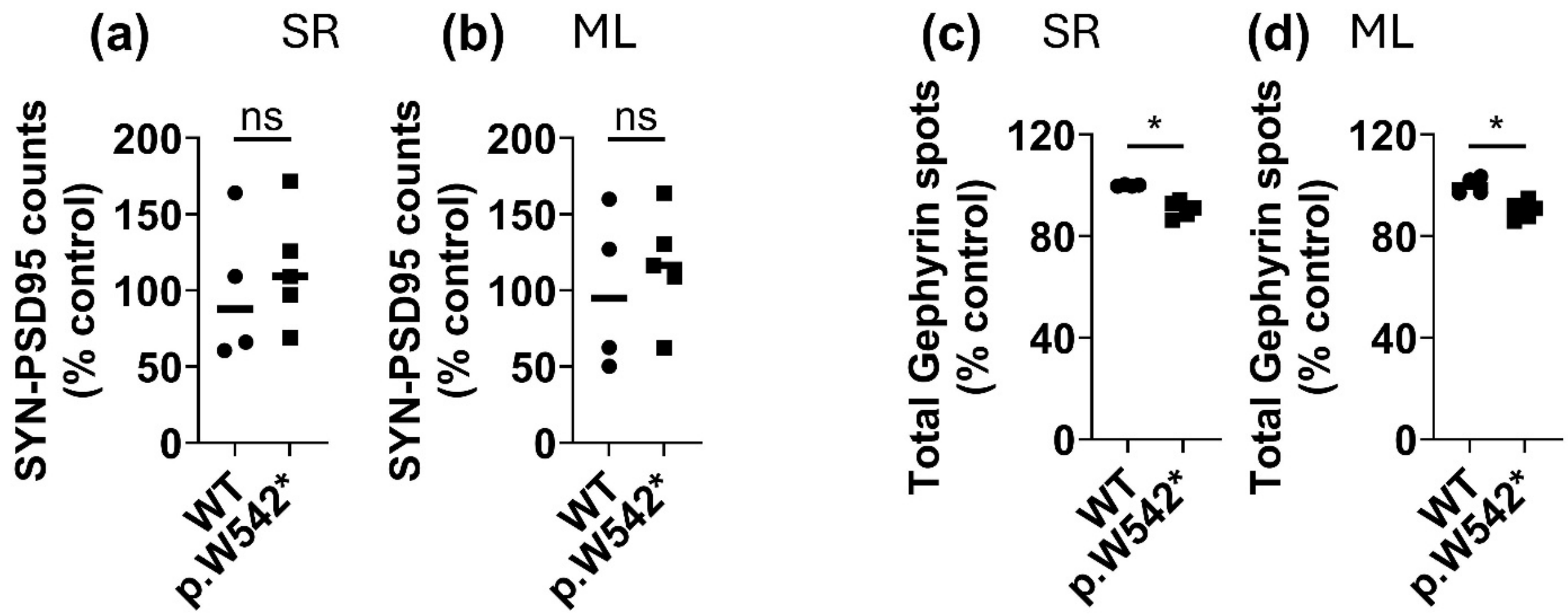

Supplementary Figure 4: Hippocampal *Wfs1* W542* reduces total gephyrin puncta in females, without a detectable reduction in excitatory synaptic loci.

(a, b) Female mice, SEQUIN quantification of colocalized synapsin–PSD-95 pairs (excitatory synaptic loci) per region of interest. Values are normalized to the wild-type group mean. (a) SR of CA1, ns: p=0.56; difference in median =+21%. (b) ML of the dentate gyrus, ns: p=0.73; difference in median =+21%. **(c, d)** Female mice, total gephyrin puncta per region of interest in SR and ML. Values are normalized to the wild-type group mean. Each symbol represents one animal, with each data point representing a mean across all ROIs imaged in that region for that animal (12–24 ROIs per animal for SR, 6-7 ROIs per animal for ML); horizontal bars indicate group medians. n=4 wild-type (WT) female mice and n=5 Wolfram homozygous (p.W542*) female mice. Statistical comparisons by Mann-Whitney test: (c) SR, p=0.016; difference in median = -8.7%. (d) ML, p=0.016; difference in median = -8.8%.

bioRxiv preprint doi: https://doi.org/10.64898/2026.09.27.754840; this version posted October 2, 2026. The copyright holder for this preprint (which was not certified by peer review) is the author/funder, who has granted bioRxiv a license to display the preprint in perpetuity. It is made available under a CC-BY 4.0 International license.

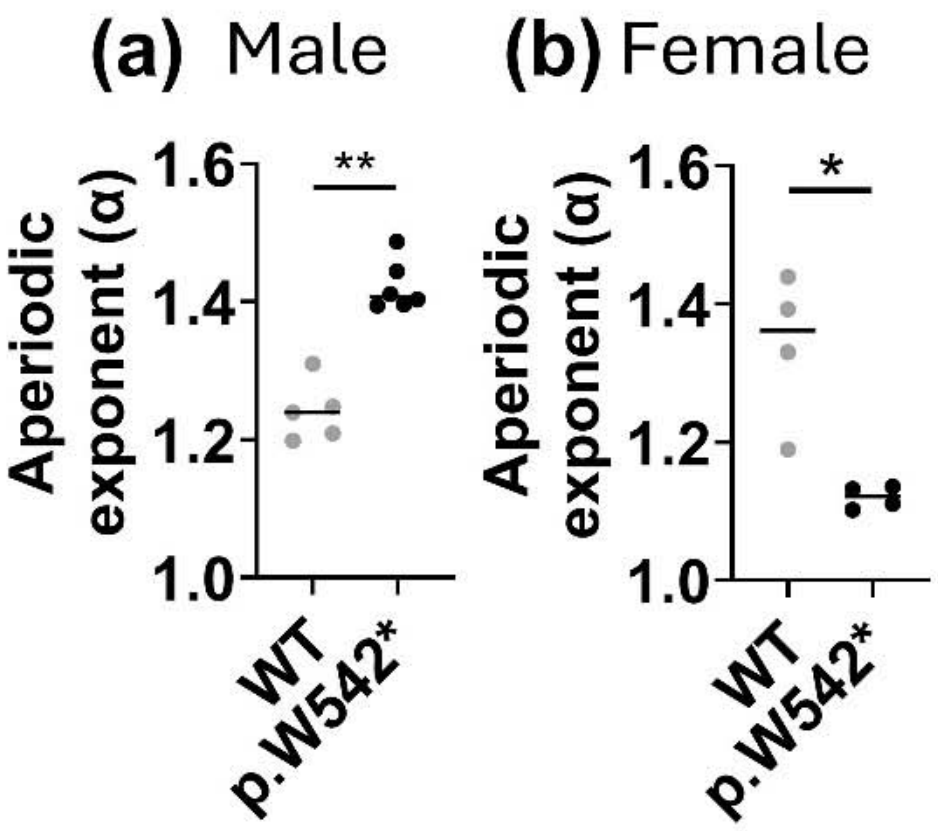

Supplementary Figure 5: Increased hippocampal aperiodic exponent in male and decreased exponent in female *Wfs1* W542* mice.
(a, b) Aperiodic exponent of the hippocampal power spectrum, fit by specparam (FOOOF – Fitting oscillations and one-over f) larger α indicating a steeper spectrum and an inhibition-dominant shift[96]. Statistical comparisons by Mann-Whitney test, (a) male **p=0.0043, difference in medians = 0.16 (WT: 1.24; p.W542*: 1.4) (b) female *p=0.028, difference in medians = -0.24 (WT: 1.36; p.W542*: 1.12). The genotype effect differed between sexes (genotype × sex interaction, permutation $p < 0.001$).

bioRxiv preprint doi: https://doi.org/10.64898/2026.09.27.754840; this version posted October 2, 2026. The copyright holder for this preprint (which was not certified by peer review) is the author/funder, who has granted bioRxiv a license to display the preprint in perpetuity. It is made available under a CC-BY 4.0 International license.

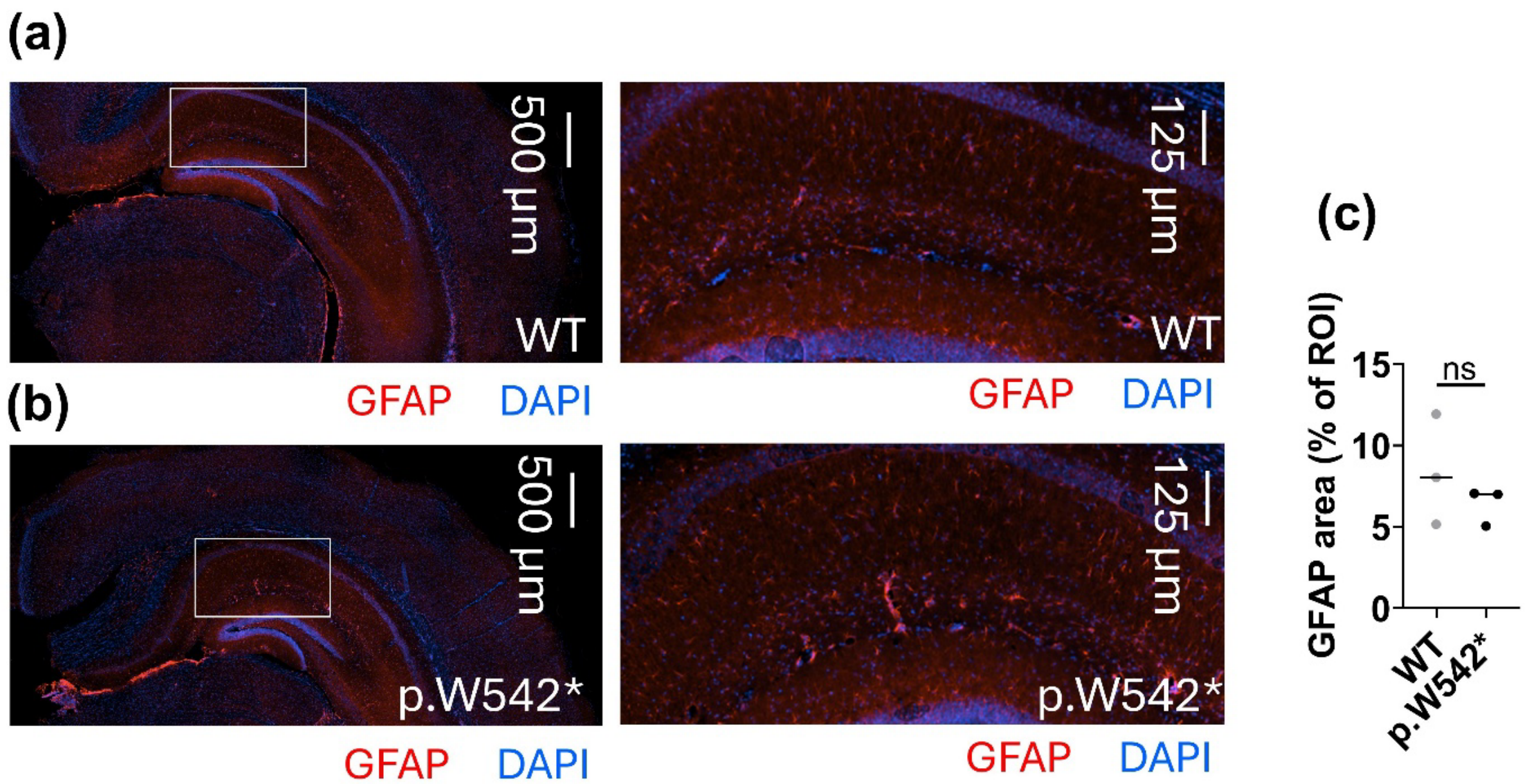

Supplementary Figure 6: Hippocampal GFAP reactivity unchanged in *Wfs1* W542* homozygous mice.

(a, b) Representative sections from (a) wild-type and (b) *Wfs1* p.W542* male mice at 18 weeks, immunostained for GFAP (red) with DAPI (blue). Left panel is a hippocampus overview with white rectangle indicating the field enlarged on the right panel, in the CA1 region of the hippocampus. Images were acquired with identical settings and are displayed with identical brightness and contrast.

(c) GFAP positive area expressed as a percentage of a fixed region of interest positioned in CA1 stratum radiatum, 390×279 µm; 108,774 μm^2, quantified in Fiji (ImageJ) from one ROI per animal. Each point represents one animal; horizontal line indicates the median. WT 8.03% (range 5.16–11.93); *Wfs1* p.W542* 6.99% (range 5.05–7.04). Exact two-sided Mann-Whitney $p = 0.40$. n=3 animals per genotype. ns, not significant. The absence of detectable astrogliosis is consistent with the absence of epileptiform activity and overt neurodegeneration in this model but does not exclude subtle astroglial changes.

bioRxiv preprint doi: https://doi.org/10.64898/2026.09.27.754840; this version posted October 2, 2026. The copyright holder for this preprint (which was not certified by peer review) is the author/funder, who has granted bioRxiv a license to display the preprint in perpetuity. It is made available under a CC-BY 4.0 International license.

Antibody	Host, clonality	Source	Catalog no.	RRID	Dilution
Primary antibodies					
Anti-synapsin 1/2	Guinea pig, polyclonal	Synaptic Systems	106 004	AB_1106784	1:1,000
Anti-PSD-95	Rabbit, polyclonal	Invitrogen	51-6900	AB_2533914	1:200
Anti-gephyrin	Chicken, recombinant monoclonal IgY	Synaptic Systems	147 009	—[b]	1:500
Anti-WFS1 (wolframin), clone EPR23801-91[a]	Rabbit, recombinant monoclonal	Abcam	ab259362	—[b]	1:1000
Anti-WFS1 (wolframin)	Rabbit, polyclonal	Proteintech	26995-1-AP	AB_2880717	1:250
Anti-NMDA Receptor 1 (GluN1)	Rabbit, monoclonal	Cell Signaling Technology	5704	AB_1904067	1:1000
Anti-AMPA Receptor 1 (GluA1)	Rabbit, monoclonal	Cell Signaling Technology	13185	AB_2732897	1:1000
Anti-β-Actin	Mouse, monoclonal	Cell Signaling Technology	3700	AB_2242334	1:1000
Anti-GFAP	Rabbit, polyclonal	Invitrogen	PA3-16727	AB_2109795	1:500
Anti-α-tubulin	Rabbit, monoclonal	Cell Signaling Technology	2128	AB_823664	1:1000
Secondary antibodies					
Goat anti–guinea pig IgG (H+L), Alexa Fluor 488	Goat, polyclonal	Invitrogen	A-11073	AB_2534117	1:200
Goat anti–rabbit IgG (H+L), Alexa Fluor 568[a]	Goat, polyclonal	Invitrogen	A-11011	AB_143157	1:200
Goat anti–chicken IgY (H+L), Alexa Fluor Plus 647	Goat, polyclonal	Invitrogen	A32933	—[b]	1:200
Goat anti–rabbit IgG (H+L), Alexa Fluor 594[a]	Goat, polyclonal	Invitrogen	A-11012	AB_2534079	1:200

Supplementary table 1: Primary and secondary antibodies used in the manuscript, with host species, clonality, commercial source, catalog number, Research Resource Identifier (RRID), and working dilution. [a] Two goat anti–rabbit secondary antibodies were used in separate labeling panels: Alexa Fluor 568 for detection of PSD-95 in the SEQUIN synaptic panel, and Alexa Fluor 594 for detection of WFS1 protein. The two panels were never combined on the same section. [b] RRID not yet assigned or not confirmed in the Antibody Registry at the time of submission.
